# Newly Developed Nano-Calcium Carbonate and Nano-Calcium Propanoate for the Deacidification of Library and Archival Materials

**DOI:** 10.1155/2017/2372789

**Published:** 2017-12-26

**Authors:** Marina Bicchieri, Federica Valentini, Andrea Calcaterra, Maurizio Talamo

**Affiliations:** ^1^Chemistry Department, Istituto Centrale Restauro e Conservazione Patrimonio Archivistico e Librario, Via Milano 76, 00184 Roma, Italy; ^2^Department of Chemistry, Università Tor Vergata, Via della Ricerca Scientifica 1, 00133 Roma, Italy; ^3^INUIT Università Tor Vergata, Via dell'Archiginnasio snc, 00133 Roma, Italy

## Abstract

Paper-based cultural heritage objects are subject to natural deterioration due to internal and external factors, that is, the presence of heavy metals, incorrect conservation, humidity, exposure of the artifacts to pollutants, light, and high temperatures. To contrast the decay of the original objects, it is necessary to study and apply innovative specific techniques, set up novel preservation methodologies, and implement or synthesize new products. As the nanomaterial science field developed over the last decades, the usage of nanomaterials in cultural heritage gained a prominent role. Such an excitement for the novel materials opened the path for an uncontrolled transfer of nanoparticles developed for different applications to paper restoration, neglecting all their possible interactions with the support or the graphic media. The aim of this work was to synthesize new nanomaterials *expressly conceived* for the treatment of library materials. To evaluate their possible insertion in the official conservation treatments that are subjected to validation by Istituto Centrale Restauro e Conservazione Patrimonio Archivistico e Librario, the new nanomaterials were tested both on laboratory paper samples and on original documents. This work presents the results of these studies (some of which still preliminary) stressing the positive and extremely promising outcomes of this research.

## 1. Introduction

Characterization of manuscripts, books, and graphic works is challenging due to the complexity of the possible interactions among all of the materials constituting the real objects: paper support, inks, dyes, sizes, and adhesives, not to mention the materials used for the bookbinding.

Each original object is unique, and no routine methods can be used to analyze them. It is necessary to study and interpret all of the possible interactions between the components of the cultural heritage object and their degradation patterns. Even though single specific problems related to the deterioration of the documents have been investigated and at times theoretically modeled for “ideal” objects, ideal materials, with single and well-defined degradation pattern, do not constitute the “real” objects. For these reasons, the techniques to be used should be carefully chosen, and the data collected with different techniques must be correctly interpreted to permit the choice of the conservation/restoration method or product to be applied to each artifact.

It is well known that oxidation and hydrolysis are the main degradation patterns that can occur in paper. Oxidation leads to the loss of paper strength and to its yellowing (by formation of carbonyl conjugated double bonds, C=C double bonds, in the cellulosic network), to the acidification of the paper (by formation of carboxyl groups), while hydrolysis induces the breaking of the chain, and the paper becomes fragile [1, 2].

Because of the specificity of the restoration treatments, it is really important to be able to distinguish the kind of degradation occurred in the paper.

In a previous study [3], we investigated, by means of Raman spectroscopy, differently degraded papers to identify the degradation products. Simply hydrolyzed papers, in which no modification of the chemical structure of the cellulose occurred, were undistinguishable from the well-conserved papers. On the contrary, oxidized papers, in which the oxidation caused chemical and structural variations in the cellulose macromolecule, showed significant differences in particular in the range below 1800 cm^−1^. It is known that the Raman intensity of some peaks depends on the orientation of the sample, with respect to the polarization of the exciting beam. Polarization analysis performed in Raman spectroscopy highlighted that several characteristic bands of the cellulose showed variations in intensity, as a function of the orientation of the fibers, but a quite broad peak at about 1577 cm^−1^ was always present in the oxidized samples and did not change in intensity with fiber orientation and rotation, making this band a good “marker” of the oxidation process.

**Figure 1:**
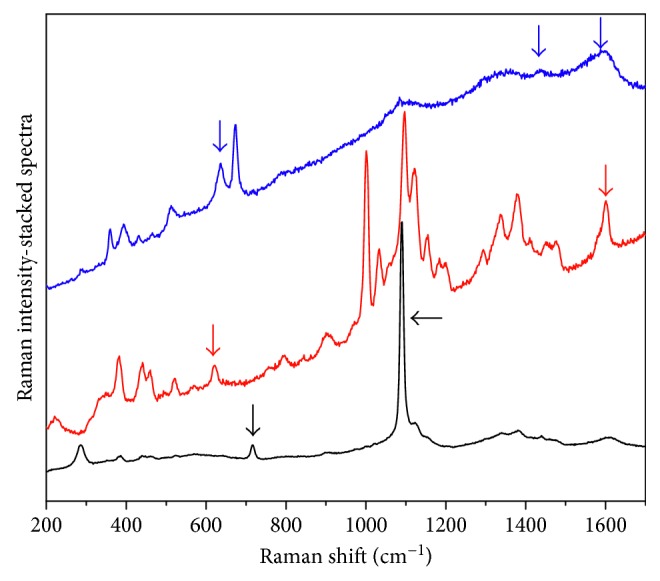
Raman spectra (excitation line *λ* = 785 nm) of papers, showing three possible degradations occurring in the cellulose chain. The characteristic peaks are marked with arrows. Blue line: formation of carboxylic groups (636 cm^−1^ O=C–O i.p. deformation; 1444 cm^−1^ O=C–O sym. stretching; 1577 cm^−1^ O=C–O asym. stretching). Red line: formation of carbon-carbon double bonds (634 cm^−1^ C=C–H wagging; 1577 cm^−1^ C=C–O stretching). Black line: formation of a five-member cyclic ether (716 C–O sym. stretching; 1079 cm^−1^ C–O asym. stretching). The ether is formed after oxidative breaking of C2–C3 bond in the anhydroglucose ring and subsequent rearrangement with formation of an ether linkage between C3 and C6 [2].

It is to underline that in the region around 1600 cm^−1^, different group vibrations overlap: for the oxycellulose, C=C symmetric stretching, C=C–O symmetric stretching, and O–C=O asymmetric stretching. The presence of other bands permits to understand and confirm the formation of specific functional groups (634 cm^−1^ for C=C–H wagging; 636 cm^−1^ for O=C–O in plane deformation; 716 cm^−1^ for C–O–C symmetric stretching of a five-member ether; 1079 cm^−1^ for C–O–C asymmetric stretching of a five-member ether; 1444 cm^−1^ for O=C–O symmetric stretching; and 1577 cm^−1^ for O=C–O asymmetric stretching and C=C symmetric stretching), Figure 1.

After detecting the kind of degradation suffered by the paper [4], the correct treatment and the most adequate solvent to be applied can be chosen.

The problems related to the oxidation of cellulose and the methods of intervention on real artifacts have been discussed elsewhere [5–8] and will not be discussed here. The products studied and their application to library and archival materials have been inserted in the official methods of the Italian Ministero dei Beni e delle Attività Culturali e del Turismo [9].

To solve the problems related to the acidification of the support, *deacidification* treatments are applied [10].

The so-called *deacidification* of paper materials consists in the neutralization of the acidic groups, that is, the carboxylic functions, by formation of water-insoluble salts. Historically, calcium carbonate has been widely used for deacidification, but this compound presents some disadvantages: it is insoluble in water (bubbling of carbon dioxide in water solution is needed to obtain the dissolution) and in other organic solvents; it is only possible to obtain solutions at low concentration (0.3 g·L^−1^) and the solution should be freshly prepared to avoid reprecipitation of the salt; long time of immersion is needed to obtain a satisfactory deacidification; moreover, aqueous treatments are not always compatible with the graphic media. The calcium salt is applied in excess and not in stoichiometric amount in order both to neutralize the acidic functions present in the paper and to deliver calcium, if new acidic functions are formed.

For cleaning, mending, and reinforcement of paper, many products have been tested—natural or artificial macromolecules or synthetic polymers [11, 12].

In the last years, some nanomaterials developed for different applications [13] have been “transferred” to paper restoration, without considering the possible negative interactions.

The aim of this work was to synthesize new nanomaterials *expressly conceived* for the reinforcement and deacidification treatment of books, archival documents, and graphic works of art, to verify their behavior after application to laboratory paper samples and original documents, and to evaluate their possible application in the official conservation treatments that are subjected to validation by the Istituto Centrale Restauro e Conservazione Patrimonio Archivistico e Librario.

## 2. Materials and Methods

### 2.1. Materials

The synthesis of CaCO_3_ nanoparticles is reported in a previous work [14], while the synthesis of calcium propanoate nanoparticles is under patent as well as the new analytical fabrication of the innovative graphene sheets.

All the reagents (Sigma-Aldrich) were of analytical grade and used as received. A Milli-Q water system was used to produce ultrapure water and all daily solutions.

For the laboratory samples, Whatman CHR-1 chromatography paper (Sigma-Aldrich) was used. The paper had been then sized with cationic modified oat starch Cerestar C^∗^05702, 10% w/v in water. Some samples were acidified by exposure to vapors of concentrated hydrochloric acid (37%; Sigma-Aldrich) for 15 minutes. These samples were used both for destructive and nondestructive analyses.

The original paper samples belonged to “*Archivio di Casa Conti-Anni 1750–1789*,” no more subjected to the survey by the Italian Ministero dei Beni e delle Attività Culturali e del Turismo. The papers, sized with gelatin, had been manufactured by using 100% flax fibers and the texts written with iron-gall ink. These samples were used both for destructive and nondestructive analyses.

A peculiar original parchment document, the *Chartula* of S. Francesco (13th century), one of the two remaining autographs of the Saint, after a first analysis campaign [15], was recently reanalyzed and treated with the new synthesized nanoparticles. The document was analyzed only by nondestructive methods.

### 2.2. pH

pH measurements (10 for each sample) were performed according to standard TAPPI T 435 om-16, by using a Hanna HI 9219 pH meter and a Hanna HI 4113 flat electrode.

### 2.3. Colour Coordinates

Colorimetric measurements were performed by means of a Minolta Chroma Meter CR22 colorimeter in the CIE *L*^∗^*a*^∗^*b*^∗^ space (6 measurements for each sample and 3 measurements for each point).

### 2.4. μ-Raman Analysis

A Renishaw InVia Reflex Raman microscope with a diode LASER at 785 nm was used for the measurements in the 100–3200 cm^−1^ range with a resolution of 3 cm^−1^. It is equipped with a 1200 lines mm^−1^ grating, a Peltier cooled deep depletion CCD (576 × 384 pixel), and objectives 20x and 50x. In this study, the power on the sample was 10 mW, the laser spot measured around 20 *μ*m^2^, and 1–5 acquisitions were performed on each sample.

### 2.5. SEM/EDX

A scanning electron microscopy/energy dispersive-X-ray analysis, Zeiss LEO1550, equipped with an Edwards Scan Coat K550X sputter coater was used for the morphological characterization of the nanoparticles. The powder of the nanostructured samples was collected on aluminium stubs and fixed with a carbon tape; the sputtering current was 25 mA and the coating time 2 min.

### 2.6. XRF

An Assing Lithos 3000 portable spectrometer with a Mo X-ray tube operating at 25 kV, 0.300 mA, with a collimator of 2 mm diameter and a Zr filter, was used to measure the effectiveness of the treatment on the original parchment document. The measurements were performed in the 0–25 keV range, with a resolution of 160 eV at 5.9 keV and acquisition time of 30 min. A long acquisition time was chosen in order to evidence also minimal modification in the concentration of the elements contained in the original document.

## 3. Results and Discussion

The application of the different nanoparticles especially synthesized to be applied on library materials will be discussed in a separate section.

### 3.1. Deacidification with Nano-Calcium Carbonate

In cultural heritage framework, CaCO_3_ has been widely used for the deacidification of paper damaged by the presence of iron-gall inks or by interaction with the external environment [16, 17]. The challenge in developing nanoparticles consists in the possibility to dissolve a consolidated good deacidifier in unusual solvents. The Tor Vergata University chemical laboratories developed a novel synthesis procedure, leading to nanocarbonates with a different morphology, if compared with the commercial products. The enzymatic approach for the new nano-CaCO_3_ provides high biocompatibility toward the end users, as restorers and conservators, and the green chemistry approach makes the process ecosustainable. Moreover, the peculiar structure obtained allows for a better efficiency in penetration into the bulk of the paper and for an improved deacidification.

**Figure 2:**
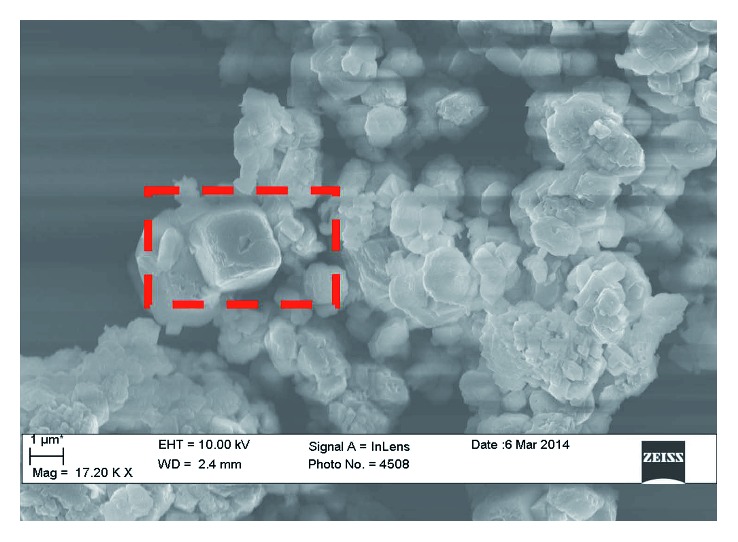
SEM micrograph of calcium carbonate nanoparticles synthesized at Tor Vergata University.

The production of CaCO_3_ nanoparticles was performed by using CaCl_2_ precursor (0.25 mol L^−1^) and the urease enzyme (at concentration of 0.25 mol L^−1^). Subsequently, urea (0.50 mol L^−1^) was added into the same solution. The onset of precipitation was highlighted by the appearance of milky clouds in the reacting solution. The obtained precipitates were filtered and washed with deionized water to remove the precursors, that is, urease and the excess of the calcium salt. In our previous paper [14], we changed and optimized the biomineralization reaction, if compared to the synthetic procedure reported by other authors [18]. The wet precipitates were immediately analyzed, under both morphological/topographic and structural point of view. The nanoparticles belong, as foreseen for calcium carbonate, to the trigonal crystal system with rhombohedral lattice and their dimensions are 9–15 nm (Figure 2). EDX spectra revealed the presence of Ca, as reported on reference [14].

Out of our knowledge, nano-calcium carbonate particles have never been applied in paper restoration prior to this study. Only nano-calcium (or magnesium) hydroxides have been proposed, but it is to underline that the official methods allowed in Italy for cultural heritage objects on paper [6] forbid the use of hydroxides, due to the too high localized pH of the compounds that can induce a ß-alkoxy elimination with subsequent depolymerization.

In the tests on original paper documents, a concentration of 3% w/v of nanocarbonate in propan-2-ol was used, and the product was applied by brushing it from the *recto* side of the documents. The penetration was good enough to avoid further treatment from the *verso* side. Preliminary results showed an absorption rate of about 0.6 g of calcium per 100 g of paper. The work is still in progress. The results, reported in Table 1, showed an increase of more than 4 pH units after the treatment both on the paper and on the acidic ink and substantially no modification in the colour.

Nano-calcium carbonate was also applied in the first documented treatment of an original ancient damaged parchment [19], to provide a calcium source, to increase the stability and the expected life of the support. The XRF measurements on the Chartula showed that the calcium content of the parchment had almost doubled after the treatment, increasing from 0.4 g Ca per 100 g of parchment before the treatment to 0.7 g Ca per 100 g of parchment after the application of nano-calcium carbonate. Raman spectra collected on the inks present on the document, that is, iron-gall ink and cinnabar, did not show any negative interaction with the graphic media.

### 3.2. Deacidification with Calcium Propanoate (Ca(CH_3_CH_2_COO)_2_)

Conservators of books, archival materials, and graphic works of art very often need nonaqueous solutions for treating original documents, to avoid the dissolution or the migration of the graphic media and the loss of the printing impression. Moreover, nonaqueous solutions allow to work on bound books, maintaining the original bindings untouched. In a research project, new methods and products were investigated to obtain an effective deacidification in a nonaqueous medium [20], and ethanol was chosen as solvent, since historical glues used in paper manufacturing are insoluble in this solvent. Calcium propanoate was tested and very good results were obtained, by treating acidic papers with a 3.5% w/v concentration. Also in this case, preliminary results showed absorption of about 0.6 g of calcium per 100 g of paper.  Table 2 summarizes the results obtained on laboratory samples treated in the alcoholic medium. Another advantage of calcium propanoate is its high solubility in water—when this medium can be used on originals—that permits to prepare the solution till 15% w/v concentration. A 10% w/v concentration in water was used—after micronization—to treat by nebulization a medieval paper containing inks soluble both in water and in ethanol. The high concentration of the deacidification solution permitted an effective deacidification (increase of 3 pH units) in a very short time (50 sec of nebulization), without any dissolution of the graphic media.

### 3.3. Deacidification with Nano-Calcium Propanoate (under Patent)

**Figure 3:**
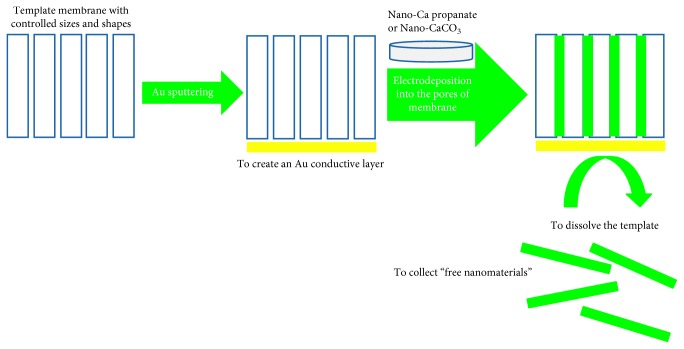
Electrochemical template synthesis approach for the nanomaterials cited in the paper.

Starting from the excellent results obtained by application of macrostructured calcium propanoate in water and ethyl alcohol solutions, it was decided to test the possibility to obtain nanoparticles of calcium propanoate. It is to underline that this approach and this synthesis were never performed before. Calcium nanopropanoate was synthesized by a new polycarbonate membrane template synthesis approach [21, 22], starting from propanoic acid in alkaline medium. The template synthesis approach can be performed following two ordered steps: conditioned-oriented chemical oxidation and electrochemical functionalization of the previously chemically obtained precursors. A scheme of the procedure is reported in Figure 3.

Also in this case, as for nano-calcium carbonate, it was possible to dissolve the deacidifier in a solvent unable to dissolve the normal calcium propanoate. In the tests on original documents, a concentration of 3% w/v of nanopropanoate in propan-2-ol was used, and the product was applied by brushing it from the *recto* side of the documents. The results obtained are reported in Table 3.

Also in this application, the penetration was good enough to avoid further treatment from the *verso* side. The results showed an increase of more than 3 pH units after the treatment of the paper and of more than 5 pH units for the acidic ink and substantially no modification in the colour, except a light increase in luminosity.

### 3.4. Reinforcement with Nanographene (under Patent—Preliminary Results)

Graphene nanosheets (3 layers: 100 nm in width and 1.017 nm in height) have been synthesized at the Tor Vergata University chemical laboratories by using two sequential mechanoelectrochemical steps. During the first step, a mechanical exfoliation of highly oriented pyrolytic graphite (HOPG) was performed by using a blender device and water or other organic solvents as the working medium. During the second step, an electrochemical oxidation occurred on the graphene sheets, mechanically detached from the HOPG layers [23, 24]. The novelty of this synthetic strategy consists in

1. two consecutive steps that guarantee a better control on the final morphology of the resulting graphene sheets. The second oxidative step allows to modulate the “surface chemistry,” essential for a selective performance on real samples;
2. the possibility to exfoliate HOPG in water, a nonconventional working medium for graphene nanoparticles, that represents an important issue for the applications needing an aqueous solution. Despite the possibility to work in water, the final system exhibits and maintains a significant hydrophobicity due to the chemical composition of graphene sheets, permitting nonaqueous applications. Preliminary mechanical tests on paper treated with nanographene gave interesting and promising results providing an improvement of its mechanical resistance. The work is in progress.

### 3.5. Raman Measurements

**Figure 4:**
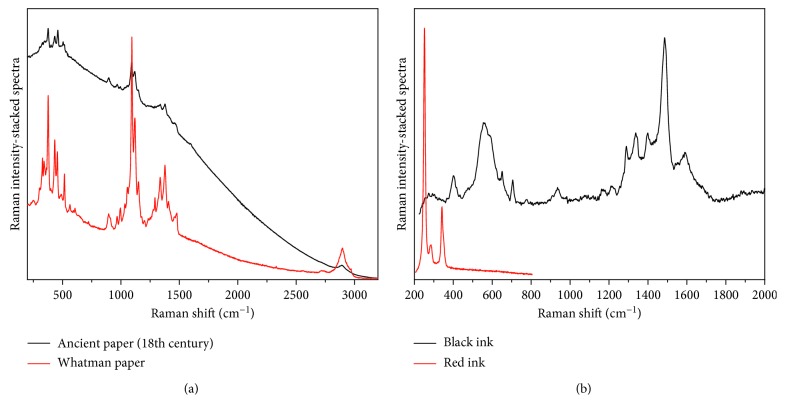
Raman spectra collected with excitation line *λ* = 785 nm. (a) Whatman paper (red line); original 18th century paper (black line) in which no signals of oxidation (marker peak at 1577 cm^−1^) are visible. The spectra are plotted without baseline correction to evidence the increase of the fluorescence background induced by the ageing in the 18th century paper. (b) Inks on the *Chartula*, that is, cinnabar (red line) and iron-gall ink (black line).

**Figure 5:**
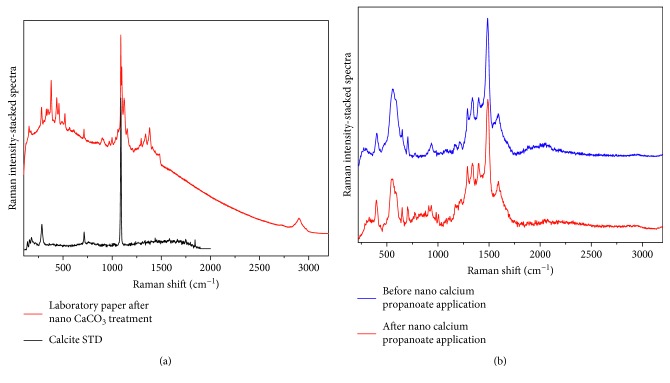
Raman spectra collected with excitation line *λ* = 785 nm. (a) Laboratory paper after the treatment with nano-calcium carbonate. The peaks related to the calcium compound, after treatment, are evidenced by comparison with a standard calcite spectrum. (b) Laboratory iron-gall ink before and after treatment with calcium propanoate. All the typical peaks of the ink (around 576 cm^−1^: band related to the formation of iron-cellulose complex; 1431 cm^−1^: COO^−^ sym. stretching, C–H and O–H bending; 1486 cm^−1^: CH_2_ scissoring, C=C ring semicircle stretching, C–H bending; 1596 cm^−1^: C=C ring quadrant stretching [25, 26]) remained unaltered after the treatment.

The paper samples, both laboratory and originals with related inks, were analyzed before and after the treatments. Some Raman spectra are shown in Figures 4 and 5.

Results showed the increase of calcium content in the paper and any negative interaction with the graphic media, in which spectra remained unaltered after the treatment.

## 4. Conclusions

In this work, the authors describe an innovative approach of conservation that comprehends the targeted application of specific techniques (i.e. *μ*-Raman spectroscopy) and the setup of new products and methods.

The results presented were obtained by using different nanomaterials, *specifically conceived* for the treatment of library materials, applied or developed at ICRCPAL Institute in cooperation with Tor Vergata University.

The newly synthesized calcium carbonate and calcium propionate nanoparticles represent a safety strategy for the treatment of ancient artworks on paper as they are not aggressive in respect to the support and the graphic media, and they can be dissolved in nonusual suitable solvents.

The new nanomaterials provide a high penetration into the deteriorated material, guaranteeing the right pH action for long time, thus conferring a long-range stability of the deacidification treatment. Moreover, the nanostructured materials are very promising because of their biocompatibility for “*end-users*” and their eco-sustainability; the latter is related to the *Green Chemistry* approach.

## Figures and Tables

**Table 1:** Treatments of original documents (18th century) with nano-calcium carbonate in propan-2-ol.

pH before treatment	pH after treatment
Paper: 6.5 ± 0.1	Paper: 10.1 ± 0.1
Ink: 5.1 ± 0.1	Ink: 9.9 ± 0.1

Colour coordinates before treatment	Colour coordinates after treatment

Paper:	Paper:
*L* ^∗^= 91.79 ± 0.12	*L* ^∗^= 90.39 ± 0.36
*a* ^∗^= +0.08 ± 0.02	*a* ^∗^= +0.19 ± 0.03
*b* ^∗^= +11.15 ± 0.18	*b* ^∗^= +10.85 ± 0.14
Ink:	Ink:
*L* ^∗^= 63.87 ± 1.63	*L* ^∗^= 69.34 ± 1.25
*a* ^∗^= +1.05 ± 0.82	*a* ^∗^= +3.02 ± 0.89
*b* ^∗^= +14.66 ± 0.54	*b* ^∗^= +13.52 ± 1.24

**Table 2:** Treatments of laboratory paper samples with calcium propanoate in ethanol.

pH before treatment	pH after treatment
Control paper: 5.4 ± 0.1	Control paper: 8.9 ± 0.1
Acidic paper: 4.9 ± 0.1	Acidic paper: 8.3 ± 0.1

Colour coordinates before treatment	Colour coordinates after treatment

Control paper:	Control paper:
*L* ^∗^= 97.15 ± 0.10	*L* ^∗^= 97.01 ± 0.08
*a* ^∗^= −0.42 ± 0.03	*a* ^∗^= −0.48 ± 0.02
*b* ^∗^= +0.68 ± 0.06	*b* ^∗^= +0.69 ± 0.05
Acidic paper:	Acidic paper:
*L* ^∗^= 96.31 ± 0.11	*L* ^∗^= 96.31 ± 0.11
*a* ^∗^= −0.80 ± 0.03	*a* ^∗^= −0.77 ± 0.03
*b* ^∗^= +2.11 ± 0.09	*b* ^∗^= +1.98 ± 0.07

**Table 3:** Treatments of original documents (18th century) samples with nano-calcium propanoate in propan-2-ol.

pH before treatment	pH after treatment
Paper: 6.2 ± 0.1	Paper: 9.5 ± 0.1
Ink: 4.3 ± 0.1	Ink: 10.0 ± 0.1

Colour coordinates before treatment	Colour coordinates after treatment

Paper:	Paper:
*L* ^∗^= 90.05 ± 0.76	*L* ^∗^= 91.05 ± 1.15
*a* ^∗^= −0.35 ± 0.02	*a* ^∗^= −0.25 ± 0.06
*b* ^∗^= +11.25 ± 0.45	*b* ^∗^= +11.85 ± 1.09
Ink:	Ink:
*L* ^∗^= 70.95 ± 0.07	*L* ^∗^= 72.05 ± 0.03
*a* ^∗^= +3.74 ± 0.68	*a* ^∗^= +2.58 ± 0.32
*b* ^∗^= +17.34 ± 0.89	*b* ^∗^= +17.87 ± 0.96
